# Causal Structure Learning Assumptions Shape Counterfactual Safety: Expert-Guided Constraints vs. Data-Driven DAGs with Probabilistic Logic Twin Networks †

**DOI:** 10.3390/e28050577

**Published:** 2026-05-21

**Authors:** Héctor Avilés, Ingridh Gracia, Rafael Kiesel, Verónica Rodríguez, Rubén Machucho, Alberto Reyes, Marco Negrete, Gabriel Ramírez, Nicolás Luévano, Myriam Pequeño, Jesús Medrano, Felix Weitkämper

**Affiliations:** 1 Department of Information Technologies, Polytechnic University of Victoria, Ciudad Victoria 87138, Mexico; 2330319@upv.edu.mx (I.G.); rmachuchoc@upv.edu.mx (R.M.); 2330215@upv.edu.mx (N.L.); 2330006@upv.edu.mx (M.P.); jmedranoa@upv.edu.mx (J.M.); 2 Institute of Computational Logic, Vienna University of Technology, 1040 Vienna, Austria; rafael.kiesel@tuwien.ac.at; 3 Computer Science Institute, Technological University of the Mixteca, Huajuapan de León 69004, Mexico; 4 Control, Electronics and Communications Department, National Institute of Electricity and Clean Energies, Cuernavaca 62490, Mexico; areyes@ineel.mx; 5 Faculty of Engineering, National Autonomous University of Mexico, Mexico City 04510, Mexico; marco.negrete@ingenieria.unam.edu; 6 Center for Research and Advanced Studies, The National Polytechnic Institute, Tamaulipas Campus, Ciudad Victoria 87138, Mexico; grtorres@cinvestav.mx; 7 Statistical and Symbolic Artificial Intelligence Group, German University of Digital Science, 14482 Potsdam, Germany; felix.weitkaemper@german-uds.de

**Keywords:** causal discovery, counterfactual reasoning, probabilistic logic, autonomous systems

## Abstract

We investigate how causal DAG learning algorithms and structural assumptions influence counterfactual decision safety. Four structure learning regimes are compared: expert-guided edge-constrained HC+BIC, unconstrained HC+BIC, MMPC+HC+BIC, and the PC-Stable algorithm. Evaluation is conducted using a leave-one-state-out protocol over a fully enumerated state–action space in a controlled offline autonomous driving setting. The environment is characterized by seven Boolean state variables and six actions, allowing us to disentangle the effects of learning strategies on counterfactual decisions. All models are implemented as probabilistic logic twin networks (*PLTNs*), with additional sensitivity analysis across parameter configurations. The learning regimes produce markedly different counterfactual decisions. Edge-constrained HC+BIC recommends a diverse set of safe actions, while unconstrained HC+BIC yields fewer but consistently safe alternatives. MMPC+HC+BIC frequently fails to identify safe actions, often associated with weak connectivity of the outcome variable. PC-Stable produces varied recommendations but may include unsafe actions, which is linked to incorrect edge orientations between actions and outcomes. These findings show that structure learning choices and prior knowledge influence counterfactual decisions through the learned structure, affecting the identification of safe alternatives in safety-critical applications.

## 1. Introduction

In recent decades, counterfactual reasoning has gained increased attention as a means of evaluating alternative scenarios by systematically altering the observed conditions and their consequences [1,2,3,4,5]. Counterfactual inferences may improve decision-making by answering actionable “*what-if*” questions, showing how outcomes could differ under alternative actions. Several approaches have been developed for this type of reasoning [6,7,8,9,10]. Among them, Pearl’s twin networks (*TNs*) [11] have been widely recognized as an expressive and principled approach to answering probabilistic counterfactual queries. TNs are built upon causal Bayesian networks (*cBNs*), whose causal structure is represented by directed acyclic graphs (*DAGs*). A DAG can be learned from observational data through a variety of approaches—for example, by incorporating structural assumptions, such as expert-guided edge constraints, and by applying score- or constraint-based learning strategies or hybrid combinations of them [12,13,14]. Despite continuous advances in the theoretical foundations of causality, the selection of reliable causal structures from data remains predominantly empirical [15,16,17,18]. On the one hand, causal structure learning and counterfactual reasoning are typically studied independently, even though they form part of the same causal reasoning pipeline: causal discovery research commonly evaluates models in terms of graph recovery [19,20,21], while counterfactual analysis often assumes a fixed underlying causal model [22,23,24,25]. On the other hand, approaches that integrate both components typically rely on a single learned or predefined causal structure and do not systematically assess how different structure learning choices or learning regimes affect downstream counterfactual decisions. Consequently, the impact of causal structure learning assumptions on counterfactual decision-making remains largely unexplored.

Therefore, this study analyzes how structural assumptions, in the form of expert-guided edge constraints on DAGs, influence counterfactual safety in preemptive decision-making. The effects of these structure learning choices are primarily assessed using the Hill Climbing (*HC*) algorithm with the Bayesian Information Criterion (*BIC*) under different settings: (i) HC+BIC with expert-guided edge constraints (hereafter referred to as constrained HC+BIC), where implausible causal associations are forbidden while guiding the search toward admissible structures; (ii) HC+BIC without structure constraints; (iii) a hybrid Max-Min Parents and Children (*MMPC*) algorithm followed by HC+BIC; and (iv) the PC-Stable algorithm without structure constraints. Regimes (ii) to (iv) represent purely data-driven DAG learning settings, in which no prior expert knowledge is incorporated. For brevity, we refer to these methods by their acronyms. The four learning approaches are evaluated offline over a fully enumerated finite scenario space of size 768, defined by seven Boolean state variables and six driving actions for a simulated self-driving car, with additional sensitivity analyses across parameter configurations. We deliberately employ a controlled state–action space to systematically compare algorithm configurations and interventions, enabling us to disentangle the effects of structure learning choices on counterfactual safety. The analysis focuses on prospective what-if questions such as “*If a self-driving car were to crash in the observed environment, which driving maneuver could reduce the likelihood of a crash?*”. This question enables self-driving cars to evaluate which alternative driving action is safer under hazardous situations. All learned DAGs are implemented through probabilistic logic twin networks (*PLTNs*) [26], which encode the probability distribution of each TN as a probabilistic logic program [27]. Probabilistic logic [28] is gaining increasing attention as a flexible and reliable knowledge representation paradigm to model probabilistic causal relationships due to its expressive and flexible rule-based representation and the availability of advanced inference procedures [29,30].

Results obtained from the main evaluation and sensitivity analysis across different parameter configurations largely confirm that structural assumptions critically shape counterfactual decision safety. Constrained HC+BIC models consistently recommend a diverse set of safe actions. This diversity may be desirable in driving scenarios, as it provides alternative maneuvers when a selected action fails to prevent a collision due to execution uncertainty or changing environmental conditions. In contrast, purely data-driven regimes exhibit heterogeneous behavior: HC+BIC produces fewer but consistently safe recommendations, whereas MMPC+HC+BIC and PC-Stable tend to recommend multiple unsafe alternatives or exhibit degenerate decision behavior, often associated with the disconnection of the outcome variable or incorrect causal orientation between the outcome and the intervention variable, respectively. These findings highlight the importance of structure learning choices, as they may affect both the learned structure and safety in autonomous driving and other high-stakes domains.

The contribution of this work is not to improve structure learning performance but to characterize how different causal learning assumptions shape the learned structure and, in turn, induce qualitatively different counterfactual decisions.

## 2. Related Work

Causal inference and probabilistic reasoning are essential for safety-critical autonomous driving challenges, particularly collision avoidance via the counterfactual evaluation of actions [31,32,33,34]. Pearl’s structural causal models and twin network semantics enable rigorous what-if reasoning about interventions in uncertain and dynamic environments, supporting explicit queries on how alternative actions affect hazardous event probabilities [35].

In system safety and reliability engineering, probabilistic counterfactual methods are used to quantify changes in risk profiles under alternative decisions. A “possible worlds” approach combines analyst-provided causal knowledge (often encoded as Bayesian network risk models) with observational evidence to rigorously evaluate hypotheses, exceeding purely associative analyses for rare, high-impact failures [36].

Reliable causal discovery is particularly crucial in high-stakes domains such as autonomous driving, where implausible structures may lead to unsafe recommendations. Reviews indicate that unconstrained score-based methods (e.g., greedy HC+BIC) can produce implausible DAGs under confounding, bias, or incomplete data coverage, motivating the use of structural constraints (e.g., blacklists, whitelists, or partial orderings) to ensure mechanistic plausibility [37]. Incorporating informed priors can improve structural accuracy, reduce spurious links, and better align learned graphs with domain knowledge [38].

In the context of traffic safety and crash analysis, Bayesian networks and causal discovery techniques have been applied to model collision likelihoods and risk factors under varying environmental conditions. Learned structures strongly influence intervention effects and the classification of safe versus hazardous maneuvers. However, prior studies typically rely either on fixed expert-designed structures or on unconstrained discovery procedures, leaving the safety implications of edge-constrained versus unconstrained or data-driven constrained learning underexplored in the context of counterfactual safe action recommendations in autonomous driving.

Recent work on probabilistic logic twin networks demonstrates that counterfactual reasoning can recommend collision-mitigating maneuvers by encoding causal Bayesian networks as probabilistic logic programs augmented with exogenous noise terms and twin network semantics [26,39]. These studies provide proof-of-concept results for interpretable, logic-based counterfactual suggestions. Nevertheless, the underlying causal Bayesian networks are typically derived either from expert design or from a single unconstrained learning strategy, leaving open the question of how different structure learning regimes systematically affect the quality and safety of counterfactual decisions. To address this underexplored issue, the present work conducts a direct empirical comparison of edge-constrained and unconstrained causal Bayesian network learning within PLTNs for counterfactual safe action selection in autonomous driving.

## 3. Learning Algorithms and Common Assumptions for Causal Structure Learning

This section focuses on a general description of the three main learning algorithms (HC+BIC, MMPC, and the PC-Stable algorithm), along with relevant aspects such as the common theoretical causal assumptions adopted in this work to infer candidate DAGs with a causal interpretation from observational data.

### 3.1. Hill Climbing and Bayesian Information Criterion

The Hill Climbing algorithm [40] performs a greedy local search to find a causal structure that optimizes a scoring function such as the Bayesian Information Criterion [41]. The BIC is a decomposable score function equal to the log-likelihood of the data given the DAG, penalized by the number of parameters. In each iteration, HC searches for the best DAG resulting from adding, reversing, or deleting edges while optimizing the BIC score. This algorithm assigns the same score to DAGs that encode the same set of conditional independencies among the variables, and it is particularly suitable for discrete data, as in the testbed described below. Other score functions include the multinomial log-likelihood (an entropy-based measure), which evaluates how well the model reproduces the observed distribution without penalizing complexity, and the Akaike Information Criterion (*AIC*) [42], an alternative to the BIC that is more commonly used in prediction tasks and typically favors denser models.

### 3.2. Max-Min Parents and Children

Max-Min Parents and Children [43] is a local causal structure discovery method. It searches for the set of directly related variables (parents and children) for a given target variable in two phases. In Phase I (forward/growth), the set of candidate parents and children (*CPC*) for the target is iteratively built by selecting, at each step, the variable whose minimum association with the target (across all possible conditioning subsets within the current CPC) is maximal. In Phase II (backward/pruning), the CPC set is refined by discarding variables that are rendered independent of the target conditioned on a subset of other candidate variables in CPC. The association between variables is measured using conditional independence (*CI*) tests such as $G^{2}$ or mutual information-based (*MI*) tests [44].

### 3.3. PC-Stable Algorithm

The PC-Stable algorithm [45] is a global constraint-based method that extends the original PC algorithm [46] for learning causal structures from observational data in an order-independent manner. It operates in two stages: (i) order-independent skeleton recovery (i.e., learning an undirected graph without edge directions) and (ii) edge orientation. The first stage starts from a fully connected undirected graph, which is systematically pruned to match the conditional independencies in the data by removing edges using CI tests. This is achieved by iteratively eliminating edges between pairs of variables that are conditionally independent given some conditioning set, with conditioning sets of increasing size. In PC-Stable, however, edge removals are delayed until all CI tests for pairs of variables with conditioning sets of size *k* have been performed. As a result, the graph does not change dynamically during testing at each level, unlike in the original PC algorithm. The separating sets identified during this process are stored for use in the next stage. In the second stage, edge directions are assigned by detecting v-structures (i.e., colliders) using the separating sets and by applying Meek’s orientation rules. The result is a completed partially directed acyclic graph (*CPDAG*), which represents the Markov equivalence class of DAGs [47] consistent with the independencies identified in the data. For counterfactual reasoning, the remaining undirected edges in the CPDAG must be oriented. This can be achieved by selecting a DAG from the equivalence class (i.e., preserving v-structures and acyclicity), by applying score-based methods such as Hill Climbing constrained to the learned skeleton, or by imposing a total ordering over variables to ensure a valid acyclic orientation.

### 3.4. Common Assumptions

Common assumptions in causal discovery include *causal sufficiency*, the *causal Markov condition*, and *faithfulness* [13]. Causal sufficiency posits that all common causes of observed variables are included in the data, ensuring no hidden confounders. The causal Markov condition (also known as the Markov assumption or local Markov condition) states that a variable is conditionally independent of its non-descendants given its parents in the DAG, enabling the factorization of the joint distribution of data. It relates the conditional independencies in a DAG (its structural properties) to the observed data distribution. Faithfulness implies that conditional independencies in the observed data distribution (numerical properties) match those implied by the DAG. These assumptions are standard, but confirming them or ensuring that they hold true is difficult in highly dynamic tasks such as autonomous driving, even in simulated (but realistic) settings, for a variety of reasons. For example, a self-driving car has to deal with many different scenarios, weather conditions, fast changes in the environment, sensor noise, or the unpredictable behavior of other vehicles. Causal sufficiency may be violated if relevant environmental variables are not represented in the DAG, such as unmodeled speed variations in the self-driving car or surrounding vehicles. The Markov condition assumes that the true data-generating distribution factorizes according to its underlying causal DAG, although this DAG is not directly observable in practice. Faithfulness may not hold due to parameter cancellation or deterministic relationships rather than structural separation. In the methodological setting proposed in the next section, causal sufficiency, the causal Markov condition, and faithfulness are treated as working assumptions rather than empirically verifiable properties. These assumptions underlie both the constraint-based algorithms used to test conditional independencies and the score-based structure learning approaches evaluated in this work. They provide the theoretical foundation for structure learning, but their degree of validity ultimately depends on the representational adequacy of the chosen state variables and the absence of significant latent confounding in the environment.

DAGs are the basis of causal Bayesian networks, which are subsequently used for counterfactual modeling, as discussed in the next section.

## 4. Methodology

This section describes the procedures implemented for learning DAGs and their corresponding cBNs and constructing the PLTNs from them. First, the simulated self-driving car is presented, from which the testbed dataset used in this work was collected.

### 4.1. Testbed and Datasets

**Figure 1:**
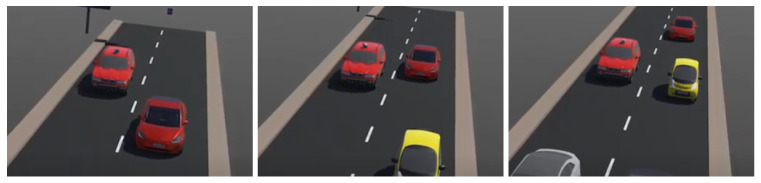
Race-like environment considered in this study for the self-driving car (bright red vehicle traveling on the right lane).

**Figure 2:**
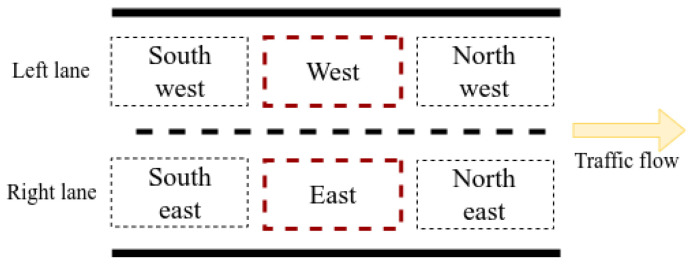
Predefined locations for other vehicles around the self-driving car (dashed red lines indicate the space that the self-driving car occupies on each lane). When the vehicle is on the left (respectively, right) lane, only the locations Northwest, Northeast, East, and Southeast (respectively, Northeast, Northwest, West, and Southwest) are meaningful.

The testbed consists of a self-driving car simulated in a race-like environment (see Figure 1). The self-driving car travels on a two-lane road with straight segments and curves and up to 10 obstacle vehicles distributed over the road, either static or moving. The maximum tested speed of the self-driving car is approximately 50 km/h. The architecture of the self-driving car includes modules for *perception*, *motor control*, *decision-making*, and *collision detection* based on radar and laser readings. State variables are curr_lane, which identifies the lane in which the self-driving car travels, and 6 occupancy variables called free_E, free_NE, free_NW, free_SE, free_SW, and free_W, which indicate whether a vehicle is present in the location specified by the name of each variable. Figure 2 depicts the locations represented by the occupancy variables on each lane. These state variables form a 7-tuple denoted as state. In addition, a multi-valued variable action identifies one of 6 driving maneuvers for the self-driving car: **change_to_left** and **change_to_right** used for changing to the left lane and changing to the right lane, respectively; **cruise** to reach a steady (maximum) speed; **keep** (distance) to maintain a steady distance to a vehicle ahead; and **swerve_right** and **swerve_left** to veer to the side of the lane while reducing the speed. The latter two actions are reserved for potential collision scenarios, and, together with **keep**, they are considered the safest actions.

Datasets were recorded under both autonomous and human control using a button-based human-friendly interface. These two datasets were integrated into a single dataset, referred to as Δ. Each possible instance from the complete state–action space of size $2^{7}\times 6=768$ was manually labeled by the co-authors as leading to a potential collision when (a) the self-driving car performed action **cruise** and there was a vehicle close ahead traveling in the same or opposite direction (rear-end or head-on collision, respectively), and (b) when there was a vehicle either adjacent to or ahead in the lane into which the self-driving car merged (sideswipe or rear-end collision, respectively). This procedure resulted in 288 state–action pairs explicitly placing the self-driving car in a potential collision scenario. All unsafe pairs involved the actions **change_to_left**, **change_to_right**, and **cruise**. Using this reference labeling, the autonomous and human control datasets contained 39,695 state–action instances corresponding to 132 distinct collision-related pairs. To ensure sufficient representation of collision cases for stable parameter estimation in the learned cBNs, the 288 distinct unsafe state–action pairs were replicated 2500 times, generating 720,000 collision instances. The number of replications was selected to significantly reduce, but not completely eliminate, the difference between safe and unsafe examples in the dataset, thereby ensuring that collision cases were sufficiently represented during learning. The resulting extended dataset is denoted $\Delta_{ext}$ and is used throughout the evaluation. All instances in $\Delta_{ext}$ were labeled using the variable latent_collision, yielding state–action-latent_collision triplets. It is worth highlighting that neither the simulated testbed nor the distribution in $\Delta_{ext}$ should be regarded as a realistic representation of real-world driving conditions. The simulator employed a race-like setting with low maximum speeds (50 km/h) and discrete states and actions precisely to isolate the decision-making module and enable a more transparent posterior analysis of the learned cBNs. Likewise, the class distribution in $\Delta_{ext}$ does not reflect the true frequency of safe and unsafe maneuvers in real traffic. Rather, both the testbed and the extended dataset constitute a controlled experimental framework designed to evaluate counterfactual decision mechanisms under balanced conditions, thereby avoiding the under-representation of rare collision scenarios. Table 1 summarizes the total number of state–action pairs labeled as potential collisions or non-potential collisions in the dataset $\Delta_{ext}$, arranged by action. Importantly, the state–action representation is treated as a fixed external constraint of the experimental framework. Thus, the objective in this work was not to optimize or redesign the state variables or action space but rather to analyze how different causal structure learning assumptions affect the learned DAGs and the resulting counterfactual decisions under the same predefined representation.

### 4.2. Learning Causal Bayesian Networks

The structure and parameter learning of causal Bayesian networks were implemented using R 4.5.3 [48] and **bnlearn** 5.1 [49]. In all cases, parameter estimation is performed using the maximum likelihood estimation (*MLE*) criterion [50], with uniform smoothing applied to child probabilities when parent configurations are unobserved. DAG learning differs across the evaluated approaches and is described in the following subsections.

**Figure 3:**
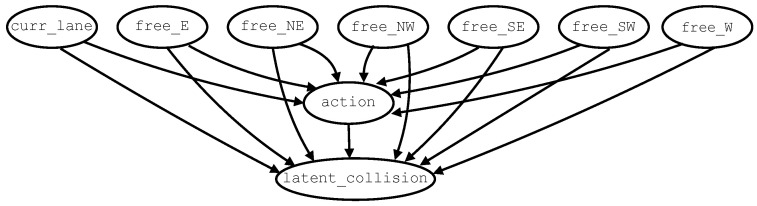
Admissible edges in causal Bayesian networks learned through (edge-)constrained HC+BIC.

HC+BIC starts from an empty structure (i.e., no edges, which is the default in many **bnlearn** configurations) and uses no restarts, resulting in a single greedy search path. The behavior of the algorithm is primarily controlled by the parameter settings restart = 0, which prevents multiple random restarts; perturb = 1, which limits perturbations to minimal local changes; maxp = ∞, which imposes no restriction on the maximum number of parents per node; and the Bayesian Information Criterion (*BIC*) as the scoring function. Expert-guided edge constraints are introduced in the form of a blacklist to forbid implausible edges in the resulting DAG. The set of forbidden edges induces a set of fifteen admissible associations, depicted in Figure 3. It is important to note that no whitelist is used during learning. In this setting, constrained HC+BIC determines which edges are included in the DAG from the admissible set and should therefore be interpreted as expert-guided structure learning rather than as a ground-truth specification. The resulting associations are considered plausible under the assumption that the observed state of the environment causally influences the driving decisions of the vehicle, which in turn affect the probability of collision. In this scheme, the variable action is treated as a treatment variable, latent_collision as the outcome variable, and the state variables as a set of Boolean confounders. The HC algorithm selects the final subset of edges from the admissible set based on the training data. As stated in Section 1, this is the only regime that incorporates expert-guided structural constraints.

Unconstrained HC+BIC employs the same parameter settings as its constrained counterpart. In this case, however, no prior assumptions regarding variable connectivity are imposed, allowing the algorithm to freely determine both the presence and direction of edges among variables.

MMPC+HC+BIC corresponds to a hybrid approach, combining constraint-based and score-based paradigms of causal discovery (through MMPC and HC+BIC, respectively). Conditional independence tests in MMPC are performed using mutual information with a significance threshold of alpha = 0.01. To control the complexity of CI testing, the maximum conditioning set size is restricted to 3 (max.sx = 3), limiting the size of conditioning sets considered during independence tests. This choice reflects a trade-off between detecting conditional independencies and maintaining the reliability of statistical estimates in finite-sample settings. MMPC produces an undirected skeleton, from which a blacklist of non-admissible edges is derived. Admissible edges are restricted to those present in the skeleton. In this way, MMPC prunes the search space of HC+BIC and serves as a data-driven approximation of plausible causal associations.

Similarly to MMPC, PC-Stable employs mutual information for conditional independence testing with a significance threshold of alpha = 0.01. In this case, no explicit restriction is imposed on the maximum conditioning set size (max.sx = NULL), allowing the algorithm to consider conditioning sets of arbitrary size. This choice increases the flexibility in detecting conditional independencies, although it may also lead to more extensive edge removal depending on the data. Once the PC-Stable algorithm has produced a CPDAG, a three-stage strategy is followed to transform the CPDAG into a DAG: (1) first, the strategy applies *cextend* [51], which orients the remaining undirected edges of the CPDAG while preserving acyclicity; (2) if *cextend* does not return a fully directed DAG, score-based refinement via Hill Climbing restricted to the learned skeleton (*HC-on-skeleton*); and (3) a fallback arbitrary orientation that preserves acyclicity based on the adjacency matrix and a topological ordering.

Although the procedures discussed above typically converge to local optima, they remain useful for identifying structural patterns that persist under resampling. In this work, stability refers to the consistency of such structural properties (particularly connectivity patterns between key variables) across different data subsets. These persistent patterns provide evidence of reliable statistical associations in the data, although they are not, by themselves, sufficient to establish causality. Accordingly, the learned DAGs should be interpreted as plausible causal hypotheses inferred from the data rather than validated causal truths.

### 4.3. Modeling Twin Networks in Probabilistic Logic

To perform counterfactual inference over the learned causal Bayesian networks, twin networks are implemented using probabilistic logic programming. The Counterfactuals package (version 1.0.0) requires as input a cBN encoded as a ProbLog program (for an introduction to ProbLog syntax, see [27]). Once a cBN is learned, as described in Section 4.2, it is translated from **bnlearn** into ProbLog syntax through a dedicated R script. During translation, the cBN is extended with *exogenous error terms* (an exogenous variable represents independent, unobserved background factors not explained within the model and is modeled as having no parents in the DAG). These error terms are introduced explicitly before constructing the twin network, since they are shared across the evidence and intervention worlds.

**Figure 4:**
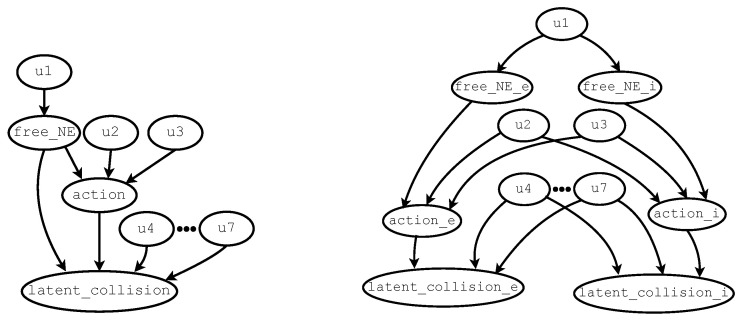
Example of a simple causal Bayesian network extended with exogenous error terms (**left**) and its corresponding twin network model (**right**). In the twin network, endogenous variables are duplicated into evidence world (“_e”) and intervention world (“_i”) copies, while exogenous variables are shared across both worlds.

A twin network model consists of two copies of a cBN. That is, both the structure and parameters of the *endogenous* variables (an endogenous variable is one that is influenced by other variables and it is defined by ProbLog rules that depend on its parent variables and associated exogenous error terms) of the original cBN are duplicated, while the two copies share the same *exogenous* error terms. The first copy represents the variables in the *evidence world*, while the second copy represents those in the *intervention world*. For illustration, consider the DAG of a cBN extended with exogenous variables as shown in the left side of Figure 4. This cBN will be used in the next subsection to illustrate the encoding of an extended cBN in ProbLog. The cBN contains only variables free_NE, action, and latent_collision. The right side of Figure 4 depicts the DAG of the corresponding twin network model obtained by duplicating the endogenous variables of the original causal Bayesian network into the evidence and intervention worlds. The suffix “_e” identifies endogenous variables in the evidence world, whereas “_i” identifies those in the intervention world. The two copies of the original cBN are linked in the twin network model through the (exogenous) error terms u1-u7. In such a model, counterfactual conditional probabilities can be evaluated, such as

(1) $$\begin{array}{cc} & p(\mathrm{latent}\_\mathrm{collision}\_i|\mathrm{latent}\_\mathrm{collision}\_e,\mathrm{free}\_\mathrm{NE}\_e,\mathrm{action}\_e,\\ & \;\;\;\mathrm{do}(\mathrm{action}\_i)) \end{array}$$

where p(·|·) is a conditional probability function; latent_collision_e, free_NE_e, and action_e are all evidence; and do(·) is the do-operator [35], which introduces an intervention over action_i. In this work, the objective is to identify a hypothetical action action_i that minimizes the probability of latent_collision_i given the current state and action.

### 4.4. Querying Counterfactual Probabilities

To answer counterfactual queries, inference in twin networks proceeds in three main steps: (a) the *abduction* step, in which the available evidence is incorporated into the evidence world to update the posterior distribution over exogenous variables; (b) the *action* step, which applies the counterfactual intervention in the intervention world; and (c) the *prediction* step, which computes the probability of the outcome variable under the intervention.

The package Counterfactuals implements these steps using a probabilistic logic programming pipeline. In this setting, a probabilistic logic program consists of *logical atoms* (i.e., Boolean variables representing events) and rules that define dependencies among them. The inference procedure proceeds as follows:

1. Assert the interventional action;
2. Construct a reduced program containing only the atoms and rules relevant to the query and evidence, represented internally as an and/or graph;
3. Ground facts and rules by instantiating all variables so that the program becomes propositional (in the present work, all atoms are already grounded);
4. Apply Clark’s completion [52], which converts the logic program into a set of logical equivalences under the *negation as failure* assumption (i.e., atoms not provable from the program are treated as false);
5. Translate the resulting program into a propositional formula in conjunctive normal form (*CNF*);
6. Compile the CNF into a *smooth deterministic decomposable negation normal form* (*sd-DNNF*) [53] using sharpsat-td [54];
7. Compute the conditional probability via *weighted model counting* [55] using the package aspmc [56].

### 4.5. A Simple Example

Recall the simplified cBN in Figure 4 presented in the previous section. The model is applied to a scenario in which the autonomous vehicle is traveling on the right lane and will observe vehicles to the left and ahead of its position. Hence, the cBN only needs to retain the variables free_NE, latent_collision, and action∈{cruise,keep}. The corresponding TN model is shown on the right side of Figure 4. The corresponding logic program, whose probabilities were learned from the dataset $\Delta_{ext}$, is described in Listing 1. Lines 3–9 declare error terms. The terms u2, u3 are binary and defined over {cruise,keep}. Annotated disjunctions (*ADs*) [57] are introduced to specify the values and probabilities for multi-valued variables in the causal model. ADs ensure that exactly one value of the variable is true at any time. In this example, error terms with ADs are defined in lines 4–5. The other error terms u1, u4–u7 are Boolean, and, for each, only its probability of being true is required. Lines 15–16 declare deterministic structural rules relating free_NE to action. For example, line 15 states that the probability of executing **cruise** is ≈0.33, while performing **keep** is ≈0.67 when the Northeast location is not empty (i.e., \+ free_NE). From line 16, it can be deduced that the preferred action is **cruise**, with a probability of ≈0.93, whenever free_NE is true (i.e., the space ahead is clear). Lines 18–21 describe the effects of action and free_NE on latent_collision. In line 18, according to the data, a potential crash is slightly more likely than not (≈0.57) whenever **cruise** is executed and there is a car ahead. Lines 19–21 and the error terms u5–u7 declared in lines 7–9 account for the low probability of a potential collision either when the action **keep** is applied or when there is no vehicle ahead. Listing 1 illustrates how the extended cBN is encoded in ProbLog syntax.



**Listing 1.** ProbLog encoding of the simple causal Bayesian network.
**1**


*%%% Error terms*




**3**

0.4618446::u1.

**4**

0.3314485::u2(cruise); 0.6685515::u2(keep).

**5**

0.9371515::u3(cruise); 0.0628485::u3(keep).

**6**

0.5765529::u4.

**7**

0.0009990::u5.

**8**

0.0838291::u6.

**9**

0.0009990::u7.



**11**


*%%% Rules*




**13**

free_NE :- u1.



**15**

action(V) :- u2(V), \+ free_NE.

**16**

action(V) :- u3(V), free_NE.



**18**

latent_collision :- u4, action(cruise), \+ free_NE.

**19**

latent_collision :- u5, action(keep), \+ free_NE.

**20**

latent_collision :- u6, action(cruise), free_NE.

**21**

latent_collision :- u7, action(keep), free_NE.



To query the corresponding PLTNs, suppose that latent_collision_e = **T** (truth value **T** means that a potential collision has been detected, while **F** means that the warning has not been raised) has been observed. It also has been observed that free_NE_e = **F** (in this case, **F** indicates that the space ahead of the self-driving car is occupied, while **T** indicates that it is empty), and action_e = **cruise**. These observations indicate that the self-driving car is accelerating forward in its lane toward its maximum speed, and there is a vehicle ahead. Finally, it is desirable to test whether a different maneuver, namely **keep**, would decrease the probability of a collision (i.e., action_i = **keep**). With these assignments, the conditional probability of latent_collision_i = **T** can be computed under the intervention **keep**, given the observed evidence. The probability of latent_collision_i = **T** is approximately 0.001, suggesting that switching to **keep** would almost certainly eliminate the risk of collision. In contrast, if the intervention sets the action to **cruise** (i.e., maintaining the same observed action under which the warning was raised), the probability of latent_collision_i = **T** becomes 1. In this case, since there is no change in the driving strategy, the possibility of a crash persists.

## 5. Evaluation and Results

In this work, a decision quality evaluation is proposed to assess whether the PLTNs learned through HC+BIC with expert-guided edge constraints, unconstrained HC+BIC, MMPC+HC+BIC, and PC-Stable select safer alternatives through counterfactual reasoning. An intervention is considered safe if it matches the manual labeling of the sample space. The sampling procedure is described next.

### 5.1. Sampling Procedure and Counterfactual Querying

For evaluation, a leave-one-state-out cross-validation (*LOSO*) design was implemented as follows.

1. Select a state–action pair from the finite state–action space (of size 768) for testing. Denote this pair as (state_e,action_e), where
  $$\begin{array}{c} \mathrm{state}\_e=(\mathrm{curr}\_\mathrm{lane},\mathrm{free}\_E,\mathrm{free}\_\mathrm{NE},\mathrm{free}\_\mathrm{NW},\mathrm{free}\_\mathrm{SE},\mathrm{free}\_\mathrm{SW},\\ \;\;\;\;\;\;\;\;\mathrm{free}\_W). \end{array}$$
2. Remove all (state,action) examples from $\Delta_{ext}$ whose state component matches state_e to prevent data leakage. This yields a smaller dataset, denoted $\Delta_{train}$, used for training and containing triplets:
  (state,action,latent_collision),
  where state≠state_e.
3. Train four independent cBNs using constrained HC+BIC, (unconstrained) HC+BIC, MMPC+HC+BIC, and PC-Stable on the same sample $\Delta_{train}$, as described in Section 4.2, and construct their corresponding PLTNs as presented in Section 4.3.
4. Append latent_collision_e=T to (state_e,action_e) to obtain the triplet
  (state_e,action_e,latent_collision_e=T).
  The assignment latent_collision_e=T simulates a potential crash in the observed state–action scenario as alerted by the forward collision warning submodule.
5. Extend the previous triplet with the variable action_i:
  (state_e,action_e,latent_collision_e=T,action_i).
6. Instantiate action_i with each value in the set
  $$\begin{array}{c} \{\mathrm{change}\_\mathrm{to}\_\mathrm{left},\mathrm{change}\_\mathrm{to}\_\mathrm{right},\mathrm{cruise},\mathrm{keep},\mathrm{swerve}\_\mathrm{right},\mathrm{swerve}\_\mathrm{left}\}, \end{array}$$
  to form a unique query group of six quartets. Each quartet in the query group shares the same components, except for the intervention in action_i.
7. Each quartet in the query group is independently presented to the three PLTNs for counterfactual querying. In each model, the goal is to identify which intervention action minimizes the probability of collision. Minimization is formalized as
  (2) $$\begin{array}{c} A_{min}^{*}={arg min}_{\mathrm{action}\_i}p(\mathrm{latent}\_\mathrm{collision}\_i|\mathrm{latent}\_\mathrm{collision}\_e,\\ \;\;\;\;\;\;\;\;\;\;\;\mathrm{state}\_e,\mathrm{action}\_e,\mathrm{do}(\mathrm{action}\_i)), \end{array}$$
  where $A_{min}^{*}$ denotes the set of one or more intervention actions action_i that minimize the probability of a potential collision within the group and for a given PLTN instance.

The above procedure is repeated for each of the 768 state–action pairs in the sample space, using the full $\Delta_{train}$ dataset as well as random subsamples of 1% and 50% of $\Delta_{train}$, stratified by action_e. All experiments were conducted on a laptop equipped with an Intel Core i5-4200U processor (4 CPU cores at 1.60 GHz) and 8 GB of RAM, running Ubuntu 24.04.3 LTS.

### 5.2. Examples of Learned DAGs

**Figure 5:**
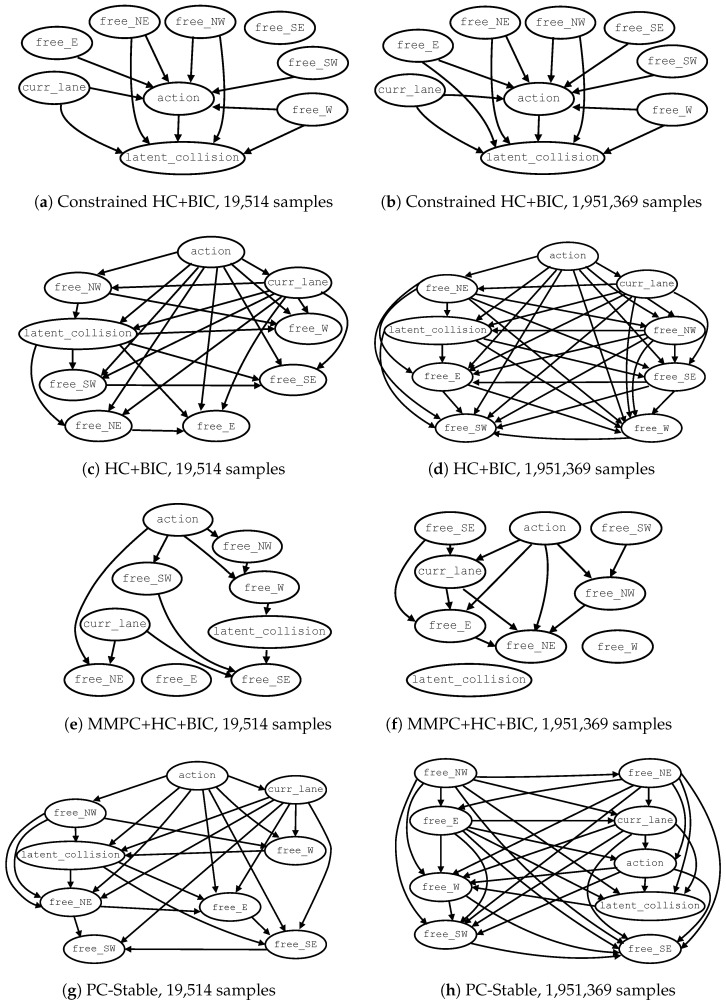
Examples of DAGs learned by each method at two training data sizes. Rows show constrained HC+BIC (**a**,**b**), HC+BIC (**c**,**d**), MMPC+HC+BIC (**e**,**f**), and PC-Stable (**g**,**h**).

**Figure 6:**
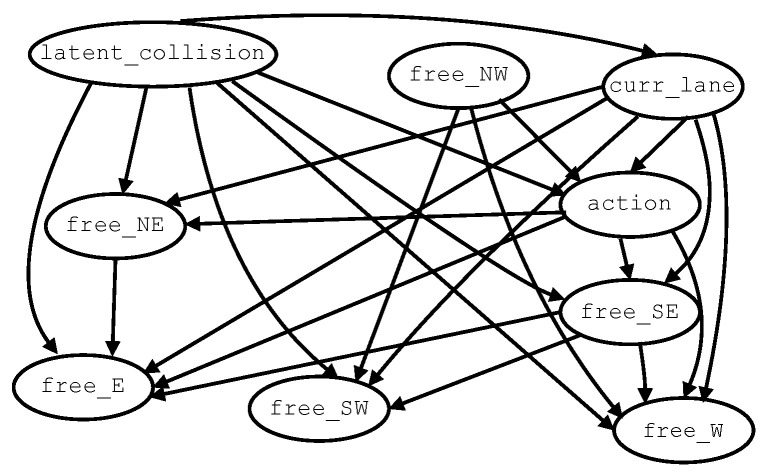
Example of a DAG with incorrect causal direction between action and latent_collision from PC-Stable.

For each learning setting, two DAGs learned from datasets of different sizes are shown in Figure 5. All DAGs were obtained from the same data samples. Figure 5a,b show the DAGs learned with constrained HC+BIC using 19,514 examples (1% of $\Delta_{\mathrm{train}}$) and 1,951,369 examples (100% of $\Delta_{\mathrm{train}}$), respectively. When training with 1%, variable free_SE is consistently disconnected from the DAG. With 50% and 100% of $\Delta_{\mathrm{train}}$, all nodes become connected. Examples of DAGs learned with (unconstrained) HC+BIC are shown in Figure 5c,d. Across all training sizes, the intervention variable action appears as the root node and is often inferred as a parent of most other variables. In this regime, several relationships are consistent with the expected causal dependencies. For instance, the edge from action to curr_lane may reflect how an action affects the lane in which the vehicle is traveling (e.g., during lane changes). These variables, together with free_NE and free_NW, frequently exhibit direct connections to latent_collision, suggesting plausible relevance for rear-end collision risk. However, other associations are difficult to justify causally. For example, edges between state variables (e.g., how occupancy in the West location could cause occupancy in the East location) lack a clear interpretation. Additional questionable relationships include edges directed from latent_collision to state variables. Figure 5e,f present DAGs learned with MMPC+HC+BIC. As with HC+BIC, some inferred associations are difficult to rationalize causally, such as those indicating the influence of free_W on latent_collision or free_SW on free_SE. Moreover, some variables may become disconnected from the learned structure. In particular, latent_collision (the outcome variable) is frequently observed to be disconnected. This behavior compromises the reliability of counterfactual decisions, as discussed in the next section. Finally, Figure 5g,h illustrate DAGs learned with PC-Stable. In most cases, latent_collision appears as an intermediate variable descendant of the intervention variable action. However, incorrect causal directions involving action are occasionally observed—in particular, when action is inferred as a child of latent_collision: (i) $\frac{37}{768}\approx 0.0481$, corresponding to 4.81% of the complete sample space with 1% of the training data; (ii) $\frac{12}{768}\approx 0.0156$, or 1.56% of the sample space with 50% of the training data; and (iii) $\frac{12}{768}\approx 0.0156$, i.e., 1.56% of the sample space with 100% of the training. One example of a DAG from PC-Stable is shown in Figure 6. Recall that the PC-Stable regime implements a three-stage strategy to complete CPDAGs into DAGs: (1) *cextend*, which orients the remaining undirected edges of the CPDAG while preserving acyclicity; (2) score-based refinement via Hill Climbing restricted to the learned skeleton (*HC-on-skeleton*); and (3) a fallback arbitrary orientation that preserves acyclicity based on the adjacency matrix and a topological ordering. At 1% of the training data, CPDAG-to-DAG completion relies on *cextend* in only $\frac{44}{768}\approx 5.7\%$ of the executions, while, in the remaining $\frac{724}{768}\approx 94.3\%$ of cases, orientation is completed using *HC-on-skeleton*. In contrast, for 50% and 100% of the training data, all CPDAGs are consistently extended using *cextend*. This behavior indicates that, under limited data, the constraint-based phase produces less informative equivalence classes, often requiring score-based refinement to obtain a valid DAG. As the sample size increases, the learned CPDAGs become sufficiently constrained to allow direct extension without additional orientation steps. Importantly, no cases required the final fallback arbitrary orientation step.

In general, these observations indicate that differences in the learned graph structures (such as disconnections of the outcome variable and incorrect causal orientations) can alter or disrupt intervention pathways, thereby affecting the reliability of the resulting counterfactual decisions, as quantified in the following section.

### 5.3. Counterfactual Safety Results Across the State–Action Space

The results for each state–action pair in the sample space are summarized in Table 2, Table 3, Table 4, Table 5 and Table 6. Table 2 reports the mean and standard deviation of the number of training samples across data percentages, along with the number of samples removed under the leave-one-state-out protocol. The mean of the number of removed examples remains constant across training percentages. This is due to the sample design, in which examples are removed before the random subsampling process to collect a percentage of $\Delta_{train}$.

Table 3 presents the performance of the four learning regimes in terms of the number of safe and unsafe intervention actions relative to the manual labeling of the sample space. The decision-making behavior is consistent across training percentages within each learning strategy. PLTN models constructed with constrained and unconstrained HC+BIC do not suggest unsafe actions at any training percentage, whereas models obtained from MMPC+HC+BIC frequently propose unsafe actions. PC-Stable also recommends unsafe actions, although to a lesser extent than MMPC+HC+BIC.

Table 4 shows the number of actions tied as optimal within each six-quartet group, along with the total number of unsafe actions. PLTNs derived from constrained HC+BIC frequently exhibit ties ranging from two to five actions, consistently excluding the observed (unsafe) action, whereas models obtained from HC+BIC propose at most two optimal (and safe) actions.

By contrast, models learned with MMPC+HC+BIC frequently generate six-way ties, particularly when 50% and 100% of the training data are used. Closer inspection reveals that this behavior is a consequence of the learned structures, in which the output variable latent_collision is disconnected from the DAG. When latent_collision is disconnected, it becomes independent of the environment state and both observed and intervention actions in the twin network, conditional on its observed counterpart. Consequently,

(3) $$\begin{array}{c} p(\mathrm{latent}\_\mathrm{collision}\_i=T|\mathrm{latent}\_\mathrm{collision}\_e=T,\mathrm{state}\_e,\mathrm{action}\_e,\\ \;\;\;\;\;\;\mathrm{do}(\mathrm{action}\_i))\\ \;=p\left(\mathrm{latent}\_\mathrm{collision}\_i=T|\mathrm{latent}\_\mathrm{collision}\_e=T\right)\\ \;=1. \end{array}$$

In this case, the value of latent_collision_i depends solely on its observed counterpart through shared exogenous variables, eliminating any influence of the intervention action. As a result, the conditional probability of latent_collision_i is identical for all intervention actions, producing the same counterfactual probability across all six alternatives and therefore systematic ties.

PC-Stable models exhibit a more varied number of optimal actions, although, in some cases, they also generate six-way ties across training percentages. This behavior arises from the incorrect orientation of the causal relationship between action and latent_collision, specifically when action is learned as a child of latent_collision. In this configuration, the intervention variable is not an ancestor of the outcome. Under intervention, incoming edges into action_i are removed, eliminating any dependence on latent_collision_i. Consequently, no directed path exists from action_i to latent_collision_i, and the intervention does not propagate to the outcome. In this case,

(4) $$\begin{array}{cc} p(\mathrm{latent}\_\mathrm{collision}\_i= & T|\mathrm{latent}\_\mathrm{collision}\_e=T,\mathrm{state}\_e,\mathrm{action}\_e,\\ & \mathrm{do}(\mathrm{action}\_i)) \end{array}$$

remains invariant with respect to the intervention. This misorientation may arise either during the constraint-based phase or during the CPDAG-to-DAG completion step. While PC-Stable identifies the skeleton and orients v-structures, the direction of the remaining edges depends on the completion procedure. In the absence of sufficient directional information, both stages may contribute to incorrect orientations, such as latent_collision → action. The resulting behavior is analogous to that described in Equation (3) and systematically produces ties in the counterfactual decisions whenever this structural condition is present. These results further show that unsafe actions are concentrated in high-order ties, particularly for MMPC+HC+BIC and PC-Stable, whereas no unsafe actions are observed for HC+BIC-based models regardless of the number of tied actions.

Table 5 reports how often each intervention action was selected as optimal. PLTNs learned from constrained HC+BIC select a broad set of actions, whereas models learned from HC+BIC primarily suggest **keep**, **swerve_left**, and **swerve_right**. Recall from Table 1 that these actions were originally labeled as safe in the dataset $\Delta_{ext}$. For models learned with MMPC+HC+BIC, optimal action tying leads to all actions being selected with equal frequency in most cases, particularly as the amount of training data increases. PC-Stable models also exhibit a broad action selection, often accompanied by the recommendation of unsafe actions.

Finally, Table 6 presents the average training and testing times required to learn the DAGs and evaluate their corresponding PLTNs for each training percentage. DAGs learned with constrained HC+BIC require lower training times due to the reduced search space induced by the edge blacklist. However, their corresponding PLTNs required higher testing times. This behavior is primarily explained by the structural position of the output variable latent_collision. Visual inspection of the learned DAGs shows that this variable typically has between five and eight parents in the constrained models, resulting in larger conditional probability tables (*CPTs*). Since each CPT entry is translated into a logic rule in the PLTN representation, larger parent sets lead to more rules, larger grounded CNF formulae, and increased weighted model counting costs during inference. In contrast, DAGs learned with HC+BIC, MMPC+HC+BIC, and PC-Stable require longer training times due to the unconstrained edge search space but often assign fewer parents to latent_collision. This yields smaller CPTs, fewer logic rules, and reduced inference complexity, explaining the lower testing times observed for these models. These structural differences are reflected in Table 7, where the number of rules defining latent_collision serves as a direct proxy for the CPT size of latent_collision and, consequently, for its inference complexity.

### 5.4. Sensitivity Analysis

The results are aggregated over 78 evaluation instances, each corresponding to a state–action pair obtained via stratified sampling on the action variable. For each instance, a LOSO protocol is applied, where all examples sharing the same state component are excluded from the training dataset, as defined in Section 5. Table 8 reports the results of the sensitivity analysis across all learning regimes and parameter configurations. Note that our goal is to analyze how the parameter configurations affect counterfactual decisions through the learned structures, rather than to optimize structure estimation. The results indicate that the main qualitative behaviors observed in the primary evaluation remain stable under parameter variations. In particular, both constrained and unconstrained HC+BIC consistently produce only safe actions across all configurations, with minor variations in the number of recommended actions. Changes in parameters such as the number of restarts, perturbation strength, maximum number of parents, or scoring function (BIC vs. AIC) have a negligible impact on the resulting counterfactual decisions. Similarly, MMPC+HC+BIC exhibits this behavior. Across most configurations, the model produces a large number of tied optimal actions, including both safe and unsafe recommendations. Variations in the significance threshold alpha and the choice of CI test (mutual information or $\chi^{2}$) have limited effects. The maximum conditioning set size (max.sx) has a more noticeable impact: increasing its value reduces the number of tied actions in some cases but does not fully resolve the presence of unsafe recommendations. A similar pattern is observed for PC-Stable. When unrestricted conditioning sets are allowed (max.sx = NULL), the number of unsafe actions is reduced, but ties still occur. Restricting the conditioning set size leads to behavior similar to that of MMPC+HC+BIC, with a large number of tied actions and increased unsafe recommendations. These results indicate that, while parameter choices influence the density of the learned structures, they do not fundamentally alter the qualitative counterfactual decisions.

To better understand these effects, Table 9 illustrates the structural role of latent_collision across seven learning regimes and parameter configurations. Constrained HC+BIC consistently places latent_collision as a pure outcome variable (Cat1) in all evaluation instances, ensuring that interventions on action propagate correctly to the outcome. In contrast, unconstrained HC+BIC consistently assigns latent_collision an intermediate role (Cat2), although without disrupting the causal influence of action. For MMPC+HC+BIC, the outcome variable is predominantly disconnected from the rest of the graph (Cat4) across most configurations, explaining the frequent occurrence of ties in the counterfactual decisions. Only in configuration C4 (max.sx = 4) does the connectivity improve, partially restoring a correct outcome role (Cat1) in a subset of evaluation instances. PC-Stable exhibits a mixture of structural roles. While some configurations produce valid structures where latent_collision acts as an outcome or intermediate variable, others frequently yield misoriented relationships (Cat3), where action becomes a child of latent_collision, preventing the intended causal influence and leading to degenerate or unreliable recommendations.

To further investigate the origins of unexpected structural behaviors, we analyze the MMPC skeletons prior to score-based refinement. For this analysis, a LOSO protocol is applied over a stratified random sample of 30 distinct state-action examples, selected based on the action variable. Table 10 shows that latent_collision is frequently weakly connected or disconnected across a wide range of parameter configurations. This behavior persists across most parameter configurations and is largely insensitive to the choice of significance level and CI test, although modest reductions in connectivity are observed in some cases when synthetic crash examples are removed. These findings indicate that the observed structural deficiencies originate in the constraint-based skeleton learning stage, rather than in subsequent edge orientation or counterfactual inference. The MI-SH test (a shrinkage-corrected variant of mutual information) was additionally considered in the skeleton analysis as part of a follow-up investigation into the structural behavior of MMPC, given the connectivity issues observed in the main evaluation.

Overall, the sensitivity analysis suggests that the qualitative differences between learning regimes are persistent under parameter variations. While hyperparameters can influence the graph density and connectivity, the dominant factor affecting counterfactual decisions is the structural role assigned to the outcome variable during causal structure learning.

### 5.5. Discussion

The results reported in the previous section highlight that structural learning choices directly shape counterfactual intervention behavior. These findings further suggest that, when the goal is counterfactual decision-making, evaluating learned causal structures solely based on the structural properties of the learned graph (e.g., edge presence and orientation, connectivity, node roles, and overall graph sparsity, as well as differences from a reference structure and goodness-of-fit to data) may be insufficient. Assessing the appropriateness of the resulting counterfactual decisions provides a complementary perspective that is directly aligned with the intended use of the model. Table 11 summarizes the main characteristics observed across the evaluated models.

Two limitations of the current methodology should be acknowledged: (a) limited scalability to more complex driving scenarios and (b) the absence of real-vehicle deployment. The approach requires the evaluation of multiple counterfactual queries across state–action combinations, each involving probabilistic inference over twin networks. As the numbers of variables, actions, and agents increase, both the size of the state space and the cost of inference grow rapidly, making the approach computationally demanding. Nevertheless, the use of controlled experiments enables the isolation of the effects of learning regimes and data distributions as sources of variation in counterfactual safety. Moreover, the PLTNs constructed in this work are not intended for real-world deployment but rather to evaluate different causal learning regimes within a counterfactual decision-making framework.

## 6. Conclusions and Future Work

Four causal structure learning strategies for constructing probabilistic logic twin networks (*PLTNs*) for counterfactual collision avoidance in self-driving cars were compared using a comprehensive end-to-end pipeline, from structure learning to counterfactual decision-making and safety evaluation. The strategies included Hill Climbing with the Bayesian Information Criterion (*HC+BIC*) with expert-guided edge constraints; unconstrained HC+BIC; a hybrid data-driven approach combining Max–Min Parents and Children with HC+BIC (*MMPC+HC+BIC*); and the PC-Stable algorithm. The results obtained from an exhaustive evaluation over the complete sample space, supported by sensitivity analyses, suggest that causal structure learning choices are not neutral: they influence counterfactual decision-making through the learned DAG, with potential implications for safety in autonomous driving systems. In particular, the results suggest that incorporating external expert knowledge through structural constraints can lead to substantially safer and more reliable counterfactual decisions in this setting. More broadly, these results indicate that structural evaluation alone may not fully capture the suitability of learned models for counterfactual decision-making. Rather than seeking to improve structure learning performance, this work characterizes how different causal learning assumptions affect counterfactual decisions and their safety implications.

As future work, we plan to extend this study by exploring additional causal discovery methods, including constraint-based approaches such as Fast Causal Inference (*FCI*), to further assess the impact of alternative structural assumptions. We also aim to conduct extended evaluations in more realistic benchmark datasets, including driving simulators and the AutoMiny V4 platform (a scaled autonomous vehicle developed at the Free University of Berlin; https://autominy.github.io/AutoMiny/, accessed on 1 May 2026), to better understand the practical implications of these structural choices for safe and efficient autonomous driving.

## Figures and Tables

**Table 1:** Total number of state–action pairs labeled as potential and non-potential collisions in the dataset $\Delta_{ext}$, categorized by action.

Action	# of Non-Potential Collisions	# of Potential Collisions	Total
**change_to_left**	40,308	289,809	330,117
**change_to_right**	39,814	294,459	334,273
**cruise**	617,930	175,429	793,359
**keep**	496,558	0	496,558
**swerve_left**	2883	0	2883
**swerve_right**	1681	0	1681
Total	1,199,174	759,697	1,958,871

**Table 2:** Mean number of training samples and samples removed due to the leave-one-state-out protocol for each training percentage (mean ± standard deviation). The same sampling procedure was applied to all models.

Training Percentage	Training Samples (Mean ± SD)	Samples Removed (Mean ± SD)
1%	19,435.75±205.92	15,303.66±20,585.24
50%	971,782.59±10,292.58	15,303.66±20,585.24
100%	1,943,565.34±20,585.24	15,303.66±20,585.24

**Table 3:** Number of safe and unsafe intervention actions selected by the different PLTN models, according to the manual “safe/unsafe” labeling of the state–action space. Counts aggregate all optimal interventions, including ties, across query groups.

Action Label	Constrained HC+BIC				HC+BIC				MMPC+HC+BIC				PC-Stable		
	1%	50%	100%		1%	50%	100%		1%	50%	100%		1%	50%	100%
Safe	1661	1731	1784		844	953	1253		603	2400	2400		984	1802	1924
Unsafe	0	0	0		0	0	0		280	2208	2208		107	37	37
Total	1661	1731	1784		844	953	1253		883	4608	4608		1091	1839	1961

**Table 4:** Distribution of the number of actions tied as optimal across learning methods. Each entry reports “tied actions/query groups” and the number of unsafe actions. Totals are aggregated across all tie levels.

Constrained HC+BIC								
# of Optimal Actions	1%			50%			100%	
	Tied/Groups	Unsafe		Tied/Groups	Unsafe		Tied/Groups	Unsafe
1	249/249	0		204/204	0		185/185	0
2	468/234	0		518/259	0		502/251	0
3	609/203	0		654/218	0		714/238	0
4	300/75	0		320/80	0		348/87	0
5	35/7	0		35/7	0		35/7	0
6	0/0	0		0/0	0		0/0	0
Total	1661/768	0		1731/768	0		1784/768	0
HC+BIC								
# of Optimal Actions	1%			50%			100%	
	Tied/Groups	Unsafe		Tied/Groups	Unsafe		Tied/Groups	Unsafe
1	692/692	0		583/583	0		283/283	0
2	152/76	0		370/185	0		970/485	0
3	0/0	0		0/0	0		0/0	0
4	0/0	0		0/0	0		0/0	0
5	0/0	0		0/0	0		0/0	0
6	0/0	0		0/0	0		0/0	0
Total	844/768	0		953/768	0		1253/768	0
MMPC+HC+BIC								
# of Optimal Actions	1%			50%			100%	
	Tied/Groups	Unsafe		Tied/Groups	Unsafe		Tied/Groups	Unsafe
1	702/702	255		0/0	0		0/0	0
2	82/41	1		0/0	0		0/0	0
3	51/17	0		0/0	0		0/0	0
4	0/0	0		0/0	0		0/0	0
5	0/0	0		0/0	0		0/0	0
6	48/8	24		4608/768	2208		4608/768	2208
Total	883/768	280		4608/768	2208		4608/768	2208
PC-Stable								
# of Optimal Actions	1%			50%			100%	
	Tied/Groups	Unsafe		Tied/Groups	Unsafe		Tied/Groups	Unsafe
1	601/601	0		136/136	0		93/93	0
2	244/122	0		620/310	0		588/294	0
3	24/8	0		705/235	0		825/275	0
4	0/0	0		276/69	0		348/87	0
5	0/0	0		30/6	0		35/7	0
6	222/37	107		72/12	37		72/12	37
Total	1091/768	107		1839/768	37		1961/768	37

**Table 5:** Frequency of counterfactual action selection across all query groups for each learning strategy and training percentage.

Action	Constrained HC+BIC				HC+BIC				MMPC+HC+BIC				PC-Stable		
	1%	50%	100%		1%	50%	100%		1%	50%	100%		1%	50%	100%
**change_to_left**	80	80	80		0	0	0		283	768	768		37	38	32
**change_to_right**	0	80	80		0	0	0		67	768	768		37	90	92
**cruise**	320	320	320		0	0	0		9	768	768		39	327	327
**keep**	558	529	539		287	287	287		92	768	768		628	592	624
**swerve_left**	353	410	435		20	98	272		319	768	768		230	497	568
**swerve_right**	350	312	330		57	64	1		113	768	768		120	295	318
Total	1661	1731	1784		844	953	1253		883	4608	4608		1091	1839	1961

**Table 6:** Mean training times of cBNs and evaluation times of their corresponding PLTNs for each training percentage (mean ± standard deviation, in seconds).

Constrained HC+BIC		
	Training time (mean ± SD, s)	Testing time (mean ± SD, s)
1%	0.03±0.01	2.54±0.07
50%	1.45±0.65	5.07±0.08
100%	10.09±5.37	4.96±0.17
HC+BIC		
	Training time (mean ± SD, s)	Testing time (mean ± SD, s)
1%	0.10±0.04	2.11±0.37
50%	3.94±1.86	2.94±0.44
100%	13.38±0.60	3.48±0.52
MMPC+HC+BIC		
	Training time (mean ± SD, s)	Testing time (mean ± SD, s)
1%	0.21±0.04	1.90±0.40
50%	6.00±0.28	1.12±0.01
100%	12.09±0.53	1.12±0.01
PC-Stable		
	Training time (mean ± SD, s)	Testing time (mean ± SD, s)
1%	0.24±0.01	2.0331±0.3977
50%	6.11±0.60	2.4440±0.4083
100%	100.97±7.50	2.3762±0.3670

**Table 7:** Mean number of logic rules and mean number of rules defining latent_collision for each training percentage (mean ± standard deviation).

Constrained HC+BIC		
	Logic rules (mean ± SD)	latent_collision rules (mean ± SD)
1%	167±0.0	96±0.0
50%	327±0.0	192±0.0
100%	327±0.0	192±0.0
HC+BIC		
	Logic rules (mean ± SD)	latent_collision rules (mean ± SD)
1%	251.99±14.62	24.50±3.32
50%	1050.75±149.58	48.38±4.23
100%	1380.50±205.28	48.37±4.23
MMPC+HC+BIC		
	Logic rules (mean ± SD)	latent_collision rules (mean ± SD)
1%	51.51±7.10	2.44±1.43
50%	100.56±4.34	1.0±0.0
100%	99.43±5.42	1.0±0.0
PC-Stable		
	Logic rules (mean ± SD)	latent_collision rules (mean ± SD)
1%	214.223±26.166	23.643±5.886
50%	850.964±80.321	115.641±42.256
100%	863.523±70.491	109.516±37.419

**Table 8:** Sensitivity analysis across all learning regimes and parameter configurations. Values are reported as mean ± standard deviation over 78 evaluation instances, each corresponding to a sampled state-action pair. The same sample was used for all configurations. Safe and unsafe actions denote the number of ranking-1 actions labeled as safe or unsafe, respectively, while total actions include all ranking-1 actions (including ties).

Constrained HC+BIC								
Config. label	Parameters							
	restart	perturb	maxp	score	Safe actions	Unsafe actions	Total actions	N
Baseline	0	1	∞	BIC	2.26 ± 1.00	0.00 ± 0.00	2.26 ± 1.00	78
C1	5	1	∞	BIC	2.27 ± 1.02	0.00 ± 0.00	2.27 ± 1.02	78
C2	10	1	∞	BIC	2.28 ± 1.01	0.00 ± 0.00	2.28 ± 1.01	78
C3	0	1	3	BIC	2.33 ± 0.75	0.00 ± 0.00	2.33 ± 0.75	78
C4	0	1	5	BIC	2.36 ± 1.10	0.00 ± 0.00	2.36 ± 1.10	78
C5	0	2	∞	BIC	2.26 ± 1.00	0.00 ± 0.00	2.26 ± 1.00	78
C6	0	1	∞	AIC	2.26 ± 1.00	0.00 ± 0.00	2.26 ± 1.00	78
HC+BIC								
Config. label	Parameters							
	restart	perturb	maxp	score	Safe actions	Unsafe actions	Total actions	N
Baseline	0	1	∞	BIC	1.14 ± 0.35	0.00 ± 0.00	1.14 ± 0.35	78
C1	5	1	∞	BIC	1.13 ± 0.34	0.00 ± 0.00	1.13 ± 0.34	78
C2	10	1	∞	BIC	1.14 ± 0.35	0.00 ± 0.00	1.14 ± 0.35	78
C3	0	1	3	BIC	1.60 ± 0.49	0.00 ± 0.00	1.60 ± 0.49	78
C4	0	1	5	BIC	1.14 ± 0.35	0.00 ± 0.00	1.14 ± 0.35	78
C5	0	2	∞	BIC	1.14 ± 0.35	0.00 ± 0.00	1.14 ± 0.35	78
C6	0	1	∞	AIC	1.14 ± 0.35	0.00 ± 0.00	1.14 ± 0.35	78
MMPC+HC+BIC								
Config. label	Parameters							
	alpha	max.sx	test		Safe actions	Unsafe actions	Total actions	N
Baseline	0.05	3	MI		3.04 ± 0.86	2.83 ± 0.76	5.87 ± 0.80	78
C1	0.01	3	MI		3.04 ± 0.86	2.83 ± 0.76	5.87 ± 0.80	78
C2	0.10	3	MI		3.04 ± 0.86	2.83 ± 0.76	5.87 ± 0.80	78
C3	0.05	2	MI		3.13 ± 0.71	2.87 ± 0.71	6.00 ± 0.00	78
C4	0.05	4	MI		1.97 ± 1.47	1.65 ± 1.36	3.63 ± 2.51	78
C5	0.01	2	MI		3.13 ± 0.71	2.87 ± 0.71	6.00 ± 0.00	78
C6	0.05	3	$\chi^{2}$		3.04 ± 0.86	2.83 ± 0.76	5.87 ± 0.80	78
PC-Stable								
Config. label	Parameters							
	alpha	max.sx	test		Safe actions	Unsafe actions	Total actions	N
Baseline	0.01	NULL	MI		2.26 ± 0.86	0.10 ± 0.52	2.36 ± 1.10	78
C1	0.05	3	MI		3.13 ± 0.71	2.87 ± 0.71	6.00 ± 0.00	78
C2	0.01	3	MI		3.13 ± 0.71	2.87 ± 0.71	6.00 ± 0.00	78
C3	0.05	2	MI		3.13 ± 0.71	2.87 ± 0.71	6.00 ± 0.00	78
C4	0.05	4	MI		3.13 ± 0.71	2.87 ± 0.71	6.00 ± 0.00	78
C5	0.05	NULL	MI		2.26 ± 0.83	0.10 ± 0.52	2.36 ± 1.08	78
C6	0.05	3	$\chi^{2}$		3.13 ± 0.71	2.87 ± 0.71	6.00 ± 0.00	78

**Table 9:** Structural role of latent_collision in learned PLTNs across learning algorithms and configurations. Cat1 denotes latent_collision as an outcome node, Cat2 as an intermediate node, Cat3 as a parent node with reversed orientation, and Cat4 as a disconnected node. Values correspond to evaluation instance counts, with percentages shown in parentheses.

Constrained HC+BIC				
Config. label	Cat1 (outcome)	Cat2 (intermediate)	Cat3 (reversed)	Cat4 (disconnected)
Baseline	78 (100.0%)	0 (0.0%)	0 (0.0%)	0 (0.0%)
C1	78 (100.0%)	0 (0.0%)	0 (0.0%)	0 (0.0%)
C2	78 (100.0%)	0 (0.0%)	0 (0.0%)	0 (0.0%)
C3	78 (100.0%)	0 (0.0%)	0 (0.0%)	0 (0.0%)
C4	78 (100.0%)	0 (0.0%)	0 (0.0%)	0 (0.0%)
C5	78 (100.0%)	0 (0.0%)	0 (0.0%)	0 (0.0%)
C6	78 (100.0%)	0 (0.0%)	0 (0.0%)	0 (0.0%)
HC+BIC				
Config. label	Cat1 (outcome)	Cat2 (intermediate)	Cat3 (reversed)	Cat4 (disconnected)
Baseline	0 (0.0%)	78 (100.0%)	0 (0.0%)	0 (0.0%)
C1	0 (0.0%)	78 (100.0%)	0 (0.0%)	0 (0.0%)
C2	0 (0.0%)	78 (100.0%)	0 (0.0%)	0 (0.0%)
C3	0 (0.0%)	78 (100.0%)	0 (0.0%)	0 (0.0%)
C4	0 (0.0%)	78 (100.0%)	0 (0.0%)	0 (0.0%)
C5	0 (0.0%)	78 (100.0%)	0 (0.0%)	0 (0.0%)
C6	0 (0.0%)	78 (100.0%)	0 (0.0%)	0 (0.0%)
MMPC+HC+BIC				
Config. label	Cat1 (outcome)	Cat2 (intermediate)	Cat3 (reversed)	Cat4 (disconnected)
Baseline	2 (2.6%)	0 (0.0%)	1 (1.3%)	75 (96.2%)
C1	2 (2.6%)	0 (0.0%)	1 (1.3%)	75 (96.2%)
C2	2 (2.6%)	0 (0.0%)	1 (1.3%)	75 (96.2%)
C3	0 (0.0%)	0 (0.0%)	0 (0.0%)	78 (100.0%)
C4	35 (44.9%)	2 (2.6%)	3 (3.8%)	38 (48.7%)
C5	0 (0.0%)	0 (0.0%)	0 (0.0%)	78 (100.0%)
C6	2 (2.6%)	0 (0.0%)	1 (1.3%)	75 (96.2%)
PC-Stable				
Config. label	Cat1 (outcome)	Cat2 (intermediate)	Cat3 (reversed)	Cat4 (disconnected)
Baseline	27 (34.6%)	48 (61.5%)	3 (3.8%)	0 (0.0%)
C1	0 (0.0%)	33 (42.3%)	45 (57.7%)	0 (0.0%)
C2	0 (0.0%)	31 (39.7%)	47 (60.3%)	0 (0.0%)
C3	0 (0.0%)	33 (42.3%)	45 (57.7%)	0 (0.0%)
C4	0 (0.0%)	33 (42.3%)	45 (57.7%)	0 (0.0%)
C5	18 (23.1%)	57 (73.1%)	3 (3.8%)	0 (0.0%)
C6	0 (0.0%)	33 (42.3%)	45 (57.7%)	0 (0.0%)

**Table 10:** MMPC sensitivity analysis with and without synthetic crash examples. Values are reported as mean ± standard deviation across 30 evaluation instances, shared across all parameter combinations to ensure comparability. The table reports the number of neighbors of latent_collision in the MMPC skeleton, the percentage of instances where it is connected to action, and the total number of edges in the learned skeleton.

Parameter			Data	# of Neighbors	% of Instances with action-latent_collision Edge	Total # of Edges in Skeleton
alpha	max.sx	test				
0.05	1	MI	with	0.33 ± 0.47	0.0 ± 0.0	7.0 ± 1.5
			without	0.17 ± 0.37	0.0 ± 0.0	6.5 ± 1.2
0.05	2	MI	with	0.67 ± 0.95	0.0 ± 0.0	12.7 ± 0.9
			without	0.00 ± 0.00	0.0 ± 0.0	12.6 ± 0.9
0.05	3	MI	with	1.00 ± 1.42	0.0 ± 0.0	22.4 ± 1.1
			without	0.33 ± 0.47	33.3 ± 47.4	21.7 ± 0.7
0.05	3	MI-SH	with	1.00 ± 1.42	0.0 ± 0.0	22.5 ± 1.1
			without	0.33 ± 0.47	33.3 ± 47.4	21.7 ± 0.7
0.05	4	MI	with	1.67 ± 2.37	33.3 ± 47.4	34.6 ± 1.0
			without	1.02 ± 0.50	33.3 ± 47.4	33.5 ± 0.9
0.01	3	MI	with	1.00 ± 1.42	0.0 ± 0.0	22.4 ± 1.1
			without	0.33 ± 0.47	33.3 ± 47.4	21.7 ± 0.7
0.10	3	MI	with	1.00 ± 1.42	0.0 ± 0.0	22.4 ± 1.1
			without	0.33 ± 0.47	33.3 ± 47.4	21.7 ± 0.7
0.20	3	MI	with	1.00 ± 1.42	0.0 ± 0.0	22.4 ± 1.1
			without	0.33 ± 0.47	33.3 ± 47.4	21.7 ± 0.7

**Table 11:** Comparison of learning strategies. Intervention blocked refers to evaluation instances (state–action evaluations) in which no directed path exists from action to latent_collision, either due to misorientation (Cat3) or disconnection (Cat4).

Comparison	Constrained HC+BIC	HC+BIC	MMPC+HC+BIC	PC-Stable
Unsafe suggestions	No	No	Yes	Yes
Presence of ties	Up to 5	Up to 2	Up to 6	Up to 6
Intervention blocked	Never	Never	Frequently (Cat3+Cat4)	Configuration- dependent (Cat3)

## Data Availability

The autonomous driving and human control datasets are available at https://www.kaggle.com/autonomousvehicle/ (accessed on 1 May 2026) under the Open Database License (ODbL) v1.0. The source code for evaluation can be found at https://github.com/hector-aviles/Counterfactuals_evaluation_2025 (accessed on 1 May 2026). Instructions for running the evaluation are available upon request.

## Abbreviations

The following abbreviations are used in this manuscript:

PLTN	Probabilistic Logic Twin Network
TN	Twin Network
cBN	Causal Bayesian Network
DAG	Directed Acyclic Graph
HC	Hill Climbing
BIC	Bayesian Information Criterion
AIC	Akaike Information Criterion
MMPC	Max-Min Parents and Children
PC-Stable	Peter–Clark Stable algorithm
FCI	Fast Causal Inference
CI	Conditional Independence
CPC	Candidate Parents and Children
MLE	Maximum Likelihood Estimation
CNF	Conjunctive Normal Form
sd-DNNF	Smooth Deterministic Decomposable Negation Normal Form
LOSO	Leave-One-State-Out
ADs	Annotated Disjunctions

MI	Mutual Information
MI-SH	Mutual Information (Shrinkage Estimator)
$\chi^{2}$	Chi-Squared Test
restart	Number of Random Restarts (HC)
perturb	Perturbation Strength (HC)
maxp	Maximum Number of Parents (HC)
alpha	Significance Level for CI Tests
max.sx	Maximum Conditioning Set Size
